# Smouldering Malignant Melanoma and Metastatic Dormancy: An Update and Review

**DOI:** 10.1155/2012/461278

**Published:** 2011-11-02

**Authors:** Gérald E. Piérard, Claudine Piérard-Franchimont, Marie-Annick Reginster, Pascale Quatresooz

**Affiliations:** Department of Dermatopathology, University Hospital of Liège, 4000 Liège, Belgium

## Abstract

The fund of knowledge regarding the versatility of presentation of MM metastases is still quite incomplete. The recent literature pertaining to the current understanding of the mechanisms underlying two special features of MM metastasis is reviewed. On the one hand, a long disease-free interval (MM dormancy) may occur before the surge of overt metastases. On the other hand, the so-called MM smouldering phenomenon refers to the condition where regional metastases wax and wane for long periods of time on restricted skin regions. It is important to emphasize that local micrometastases often predict sentinel lymph node involvement but may not reflect progression of the primary MM to full-blown visceral metastatic competence. It is likely that a combination of factors impacts the versatile MM metastasic progression. Among the main factors, one has to mention the phenotypic heterogeneity and variability in the phenotype of MM cells, the presence of MM stem cells and MM cells engaged in an amplification proliferation pool, as well as the host immune response, and possibly the induction of a particular stromal structure and vascularity.

## 1. Introduction

Limitations in the understanding of the biologic versatility of malignancies including malignant melanoma (MM) have resulted in different definitions of even their most fundamental terms [1, 2]. In spite of semantic quandaries, MM is regarded as a single or mixed population of abnormal melanocytes demonstrating temporally unrestricted growth preference over the normal cell contingent. MM progression corresponds to sequential focal changes in the neoplastic cell population. These events are present from tumor induction to full blown MM including metastasis. This condition is accompanied by growth disorganisation and frequent cytologic atypia. The neoplastic melanocytes invade surrounding tissues, and some are prone to metastasize at distant sites. This process leads to a series of qualitatively different tumoral deposits. 

The evolution of MM metastases is typically the result of tumor progression and their aspects are related to (a) the malignant cell proliferation kinetics, (b) the antigenic differentiation of the cell molecular components, and (c) the release of growth factors influencing the peritumoral stroma including vascularization.

## 2. MM Growth Rate and MM Stem Cells

On clinical ground, several groups of patients may be identifiable according to the MM evolution. A first group encompasses primary MM remaining localized for months or years (slow growing MM). A second group is formed by MM growing rapidly in a matter of weeks (fast growing MM) [3]. A third group is formed by completely regressed primary MM leaving regional metastases (orphan metastases).

On histopathologic ground, the MM growth rate is related to the proliferative activity of its cells. The mitotic rate [4, 5], the S-phase index [6] and the growth fraction represented by the MIB/Ki-67 index [3, 7–11] are distinct but somewhat correlated parameters. Globally, a high proliferative activity in the primary MM irrespective of its thickness predicts early metastases to the regional lymph nodes and beyond [6–8].

The numeration of mitotic figures, being either below or above 1/mm^2^, is advocated by the American Joint Committee on Cancer (AJCC) group [5]. Unless using historne immunohistochemistry, some mitotic figures may be difficult to identify with confidence, and they may be confused with apoptotic nuclei. Another problem is linked to the precise areas to be considered in the evaluation. The difficulty is more prominent in thin MM with jagged borders. The percentage of Ki67+ nuclei show a much larger range of values among MM, and thus it is more easy to handle for discriminating the high and the low risk MM [10, 12].

The balance between cell proliferation and apoptosis is of paramount importance in the determination of the MM tumorigenic potential [13]. The proliferative activity of MM is in part regulated by its tumor vascularity [3, 13–15]. The influence of the nonvascular extracellular matrix on MM progression should not be neglected [16, 17].

The putative role of MM stem cells is increasingly recognized in the primary MM and its metastases [18–22]. In general, the antigenic patterns inside primary MM are variable and heterogeneous [12]. Some markers of melanocytic stem cells including CD166, CD133, and nestin are present in MM [20]. Such immunoreactivity likely corresponds to genetic pathways instrumental to stem cell biology. Interestingly enough, MM stem cells have a slow proliferative rate. Thus, they may remain silent for very long periods of time before initiating an amplification proliferative pool of MM cells revealing overt metastases [3, 11].

## 3. MM Progression and the Host

MM originating from the skin and subsequently releasing metastases exhibits some pathologic attributes in relation to the host. In its early stage of evolution the neoplasm does not set up any effective and complete immunologic rejection by the host, otherwise any incipient MM would be readily destroyed. In fact, the MM cell phenotype is typically heterogeneous [12] although it looks uniform at the standard histopathologic examination. With progression of the neoplasm, any mutant MM cell deprived from the innate ability to survive and escape the host defences would be readily destroyed. This process is responsible for partial MM regression which is so frequent in superficial spreading MM, occurring either spontaneously or induced by various forms of immunotherapies [23].

The process of metastasis consists of a series of linked, sequential steps. Although some of the steps in this process contain stochastic elements, metastasis as a whole favors the survival and growth of a few subpopulations of cells that preexist within the primary neoplasm. Metastases may have a clonal origin, and different metastases possibly originate from the proliferation of few cells. The outcome of metastasis depends on the interaction of metastatic cells with various host factors. Organ-specific metastases have been demonstrated in a variety of neoplasms and may be specific to a particular site within a given organ. Clonal analysis of human MM revealed that these neoplasms were heterogeneous for metastatic properties and that growth in the environment of specific organs is selective. These findings suggest that systemic physiologic signals are potentially recognized by neoplastic cells, presumably by mechanisms similar to those shared by their normal cell counterparts. 

A fundamental prerequisite for MM metastases resides in the ability of MM cells to dissociate from the primary MM and to breach a series of sequential structural and functional barriers. The progression of any primary MM and its metastases has to be adaptable to distinct and variable environments in order to survive. In all likelihood, a natural selection of MM cell phenotypes occurs during MM invasion. The same feature operates in every collection of metastatic MM cells lodging at different body sites [24]. Indeed, the local immune mechanisms may detect and destroy some metastatic cells. Meanwhile, other metastatic cells without the same attributes survive. Just as the primary MM is likely polyclonal, so are the metastases [1].

Any local recurrence at the site of the primary MM rarely develops simultaneously with disseminated disease. MM metastases commonly involve body sites where other cancer metastases are collected, namely the skin, lymph nodes, and lungs.

The host defences do not apparently achieve the same efficacy in every tissue. Therefore, it comes about that selective survival of MM cells is possible in some restricted organs and tissues. In addition, the host defences may be altered by immunosuppression [25]. An alternative possibility relies on the nature of the stroma where metastatic cells are stuck. Indeed, the stroma abutted to the primary MM exhibits a peculiar composition [16, 17] that may favor or be necessary for the neoplastic survival and growth. If these conditions are not met at the final destination of the metastatic cells, they may fail to develop overt metastasis. 

In a global perspective, the induction of a local micrometastatic process does not ineluctably lead to overt metastases at distance. Indeed, at the onset, the micrometastasis may be destroyed, remain quiescent even for years, or may grow under a positive proliferation-apoptosis balance. Typically, the fate of metastatic MM cells varies in time. Even after a long period of quiescence, overt metastasis may appear [25]. They may alternatively enter a spontaneous regression phase. In some instances, metastases appear in crops, and they grow in concert as if they were synchronized by a systemic control. In these instances, their sizes are rather similar at any time in their evolution. In other circumstances, metastases appear to grow independently each other. At the extreme, some grow while others regress in the same time. This feature has been called the MM smouldering phenomenon [26].

## 4. MM Microsatellites

Metastasis is defined as a malignant neoplasm arising from a primary or metastatic malignancy without remaining no longer in contiguity with the initial tumor. In the early step of evolution, MM lesions lack competence for metastasis. Indeed, selected metastatic steps depend on (a) the rate of cell production in the primary neoplasm, (b) the number of cells migrating away from the primary site, (c) the number of cells entering a venule or a terminal lymphatic, (d) the number of cells surviving in the blood or lymph vascular pathway, (e) the number of cells escaping from vascular pathway, and (f) the number of cells surviving and replicating at the metastatic site. Any step in the metastatic pathway must ultimately be related, in some way, to the rate of production of the cells in the primary MM [1, 4, 9, 10].

**Figure 1:**
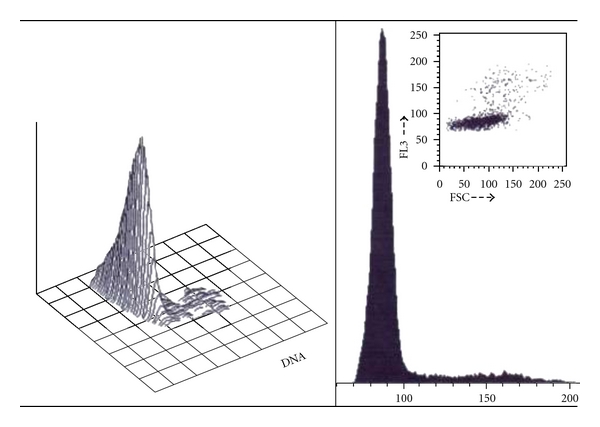
Example of DNA flow cytometry in a MM metastasis showing a variable content in cell DNA.

Presence of MM microsatellites is associated with increased overt locoregional metastases [27–30]. It may represent a significant negative predictor for relapse-free survival. By contrast, microsatellites were variably reported to decrease [28] or not [30] the overall survival. MM microsatellites appear to be intimately tied to other markers of MM aggressiveness. This concept was raised for intradermal metastases greater than 0.05 mm in diameter [27]. With the introduction of immunohistochemistry, smaller lesions were possibly identified down to single cell MM micrometastases [29]. Flow cytometry searching for DNA content in MM metastases reaching a millimetric volume variably reveals the presence of a combination of diploid, tetraploid, and various other polyploid cells (Figure 1). 

A fundamental understanding of mechanisms involved in MM metastasis has been improved over the past two decades. Several migration paths for MM micrometastases were identified in the skin. These cells may be found inside some vessel lumina [31], inserted within vessel walls [32–34], abutted to the outer portion of endothelial cells [14, 35–39], and dispersed inside the stroma [14, 29].

By definition, cells in a distant metastatic site have full metastatic competence. Consequently, it seems likely that metastasis from metastasis is a routine event in neoplastic biology. However, the role of the stroma hosting MM metastasis is not harmless because a cardinal property of a malignancy is the ability to grow in the mesenchyme at the primary and secondary sites. Indeed, the premetastatic mesenchyme is noticeably different from the normal. When neoplastic progression is active, MM lesions have both a neoplastic parenchyme and a neoplastic mesenchyme.

As the mesenchyme of the distant site is unprepared and may require a long period of neoplastic adaptation prior to significant growth. Thus, metastases may be limited to a single organ or tissue for a certain period of time. Such patterns of spread may reflect the existence of cells only capable of growth in the mesenchyme specific to a given organ or tissue. Common restricted pathways include skin metastases.

An eventual lack of metastasis could be explained by a subset of tumors capable of growth in the stroma at the primary site, but incapable of completion of any step in a metastatic pathway, except for invasion and some motility in the dermis. According to such hypothesis invasiveness and metastatic development would not be acquired in concert, but as successive cell adaptations.

## 5. Smouldering MM

The interrelations between the host and MM cells are under the influence of a vast array of factors. These events are probably not static, but result from a continuous fluctuating balance between two living systems engaged in natural selection. The changes in time of the combination of different cell processes including MM and its microenvironment may lead to a condition named smouldering MM.

The concept of smouldering MM was raised after an extensive study of MM over more than 20 years, emphasizing the way by which established metastatic disease may vary in its evolution [17]. The seminal paper described the smouldering MM phenomenon as metastases appearing and disappearing on the same body region over a period of months or years. The metastases wax and wane in an apparently haphazard manner, usually reaching at the most the size of a pea or a bean. 

The smouldering MM phenomenon contrasts with the more common synchronized burst of metastatic crops. This latter condition has been particularly observed following excision of a primary MM at a time when the metastatic diseases remained undisclosed. The smouldering MM highlights the fact that tumor progression is not ineluctable. Indeed, the net directionality of MM metastasis early in tumor progression may be toward growth or regression. This may be due to the apoptosis prevalence over proliferation, the versatility of antitumoral immunity, the lack of stromal receptivity, and any defect in angiogenesis. Of note, smouldering inflammation in the neoplasm microenvironment promotes proliferation and survival of malignant cells, angiogenesis, metastasis, subversion of adaptive immunity, as well as response to hormones, and chemotherapeutic agents [40]. This multifaceted process possibly ends in a programmed pathway of apoptosis and necrosis.

The smouldering MM phenomenon probably involves a combination of distinct cell properties related to site-specific MM cell growth, site-specific adhesion molecules, site-specific metastatic cell invasion, and site-specific regulation of metastatic cell growth and regression. Novel concepts regarding early seeding of metastases coupled to parallel progression, self-seeding of primary tumors by circulating neoplastic cells, and the induction of premetastatic niches in distant organs by primary cancers have come to the fore [41]. Such complex features have been compared to the development of plant selling [38]. Although many metastatic neoplasms are able to colonize a wide variety of tissues, the smouldering MM phenomenon frequently and almost exclusively occurs in the skin area between the primary MM and the first group of drainage lymph nodes. Hence, there are regional influences in the metastatic colony formation ruling the MM smouldering diseases. The metastatic process appears to be in a cleft stick.

## 6. MM Dormancy

Any unusually long latency period between the primary MM treatment and metastatic occurrence corresponds to a clinically disease-free condition. It is commonly thought to represent clinical MM dormancy. The relationship between such condition and the cause of MM cell dormancy is complex and probably multifactorial [42]. The process is not stable and may lead to relapse [43].

Tumoral dormancy and autophagy may be in part correlated. Autophagy is a homeostatic and catabolic process that enables the sequestration and lysosomal degradation of cytoplasmic organelles and proteins. Such process is important for the maintenance of genomic stability and cell survival. Autophagy is a mechanism of stress tolerance that maintains cell viability and possibly leads to tumor dormancy, progression, and therapeutic resistance [44].

Delay of MM metastases is a manifestation suggesting a host defence mechanism or a peculiar nature of nonproliferative MM cells possibly involving MM stem cells. Globally about 40% of patients who develop MM metastases do so more than 5 years after primary treatment. A typical example is the appearance of metastases in the liver, many years after removal of the eye affected by intraocular MM. The disease-free interval before metastases may be as long as 25 years. When metastases from uveal MM develop, they almost always appear first in the liver and often are found only in the liver. This strong tendency to involve the liver early in the course of the disease is not explained on the basis of any anatomic or physiologic factors. The reasons for the long interval from primary treatment to metastases in uveal melanoma are completely unknown. Since the eye has no lymphatic drainage, the MM must have already metastasized prior to the enucleation, and yet a long period of time may elapse before these metastases appear clinically in the liver. The rate of growth of these overt metastases varies considerably once clinically detectable. It is tempting to speculate that these metastatic implants remain dormant for many years because of some intrinsic controlling mechanism of the MM cells themselves or a systemic control by the host. 

Another example of MM dormancy deals with MM developed from transplant organs in patients placed in immunocompromised condition [24, 45, 46]. Micrometastases that had remained silent in the donor subject develop in an uncontrolled brisky way when the invaded organ is transplanted in the immunocompromised recipient.

## 7. Conclusion

The development of MM metastases is not the expression of a single uniform process. Many factors linked to the nature of the neoplastic cells and the reactivity of the host interfere in a complex way. Smouldering MM reflects the individual life of each metastasis confined to a restricted skin territory. The lesions wax and wane in an apparently haphazard and uncoordinated way. MM dormancy refers to a condition where overt metastases appear after an extended lag time. The intervention of metastatic MM stem cells or of metastatic MM cells blocked in the cell cycle of proliferation is possible.

The two phenomena, that is, the smouldering and the dorman processes presently described in the MM metastatic progression might in addition possibly occur at the primary site. The smouldering primary MM phenomenon could correspond to the partial MM regression which is a frequent observation. The primary MM dormancy would correspond to a lengthy duration between MM initiation and the early clinical stage of MM duration.

These two conditions are worth studying because new therapeutic advances could emerge by stirring up and controlling the smouldering and the dormancy MM phenomena.
